# Trans trochanteric approach with coronal osteotomy of the great trochanter: A new technique for extra-capsular trochanteric fracture patients treated by total hip arthroplasty (THA) in elderly

**DOI:** 10.1051/sicotj/2015015

**Published:** 2015-06-05

**Authors:** Francois Steffann, Jean-Louis Prudhon, Jean-Marc Puch, André Ferreira, Loys Descamps, Régis Verdier, Jacques Caton

**Affiliations:** 1 Clinique des Cèdres 21 rue Albert Londres 38432 Echirolles France; 2 Clinique Saint-George 2 Avenue de Cimiez 06100 Nice France; 3 Clinique du Parc 155 Ter Boulevard de Stalingrad 69006 Lyon France; 4 Groupe Lépine 175 rue Jacquard CS 50307 69727 Genay Cedex France; 5 Clinique Emilie de Vialar 116 rue Antoine Charial 69003 Lyon France

**Keywords:** Femoral neck fracture, Total hip arthroplasty, Hip surgical approach, Dual mobility cup, Extra capsular trochanteric fracture

## Abstract

Several surgical approaches could be used in hip arthroplasty or trauma surgery: anterior, anterolateral, lateral, posterior (with or without trochanterotomy), using or not an orthopedic reduction table. Subtrochanteric and extra-capsular trochanteric fractures (ECTF) are usually treated by internal fixation with mandatory restrictions on weight bearing. Specific complications have been widely described. Mechanical failures are particularly high in unstable fractures. Hip fractures are a major public health issue with a mortality rate of 12%–23% at 1 year. An alternative option is to treat ECTF by total hip arthroplasty (THA) to prevent decubitus complications, to help rapid recovery, and to permit immediate weight bearing as well as quick rehabilitation. However, specific risks of THA have to be considered such as dislocation or cardiovascular failure. The classical approach (anterior or posterior) requires the opening of the joint and capsule, weakening hip stability and the repair of the great trochanter is sometimes hazardous. For 15 years, we have been treating unstable ECTF by THA with cementless stem, dual mobility cup (DMC), greater trochanter (GT) reattachment, and a new surgical approach preserving capsule, going through the fracture and avoiding joint dislocation. Bombaci first described a similar approach in 2008; our trans fractural digastric approach (medial gluteus and lateral vastus) is different. A coronal GT osteotomy is performed when there is no coronal fracture line. It allows easy access to the femoral neck and acetabulum. The THA is implanted without femoral internal rotation to avoid extra bone fragment displacement. With pre-operative planning, cup implantation is easy and stem positioning is adjusted referring to the top of the GT after trial reduction and preoperative planning. The longitudinal osteotomy and trochanteric fracture are repaired with wires and the digastric incision is closed. This variant of Bombaci approach could be use routinely for hemiarthroplasty or THA in the cases of unstable ECTF. It reduces complications usually linked to this procedure. Blood loss, operating time, and pain are limited, allowing fast recovery in order to decrease morbidity and mortality.

## Introduction

**Figure 1.**
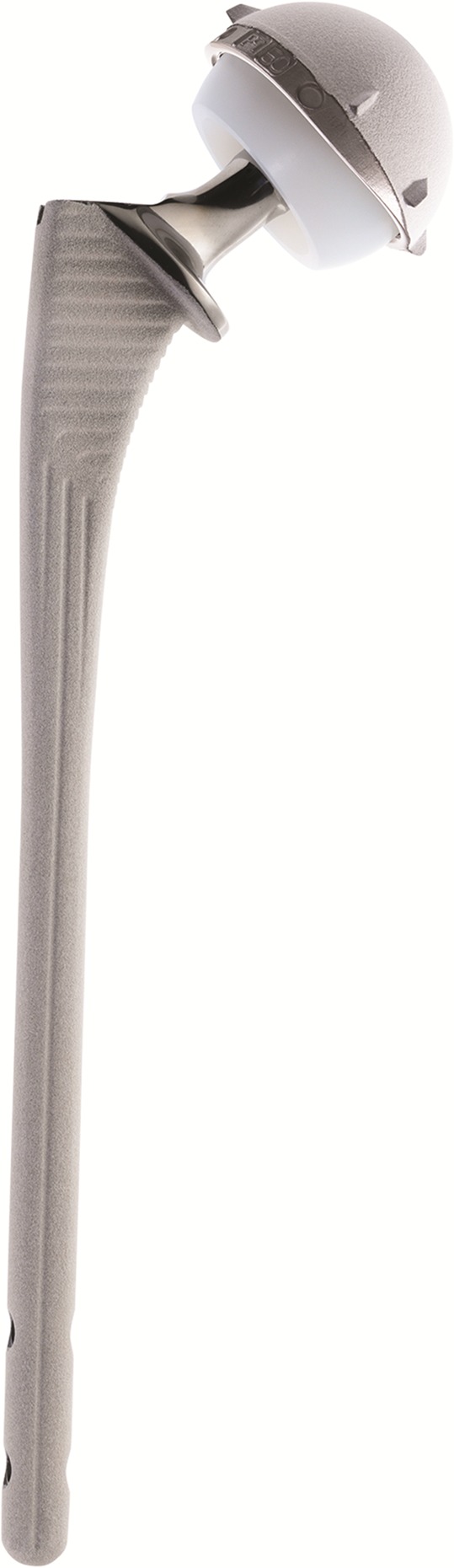
Long cementless stem TARGOS™ and DMC QUATTRO™ – groupe Lépine (Genay – France).

Trans trochanteric approach described initially by Charnley [1] for THA in degenerative hip disease has been used in France for more than 50 years with very good results at more than 25 years follow-up as shown by both senior doctors [2]. In 2008 Bombaci [3] described a trans trochanteric approach in intertrochanteric femur fractures but without greater trochanter (GT) osteotomy. For 15 years we have been using a new modified approach (trans trochanteric or trans fracture) to perform cementless THA (Figure 1) in the treatment of extra-capsular trochanteric fractures (ECTF).

Our modification is a routine GT coronal osteotomy, described in this study with the tips and tricks to implant a total hip arthroplasty (THA). It allows hip reconstruction without posterior dislocation and easy GT fixation. Goals are to recover as soon as possible mobility and independence. Outcomes depend on timing of surgery (less than 48 h), quick rehabilitation, and precise surgical technique (osteosynthesis or arthroplasty). Today, there is a trend to manage proximal femoral fracture by THA rather than hemiarthroplasty. However, ECTF or less frequently intra-capsular fracture (ICF) is complex and often associated with degenerative joint disease justifying the use of THA.

## Approach description

As often as possible, a preoperative planning on the opposite side is performed to determine hip center, implant size, and to ensure an equal leg length. After general or spinal anesthesia the patient lies in a strict lateral decubitus position on a standard operating table.

### Exposure

**Figure 2.**
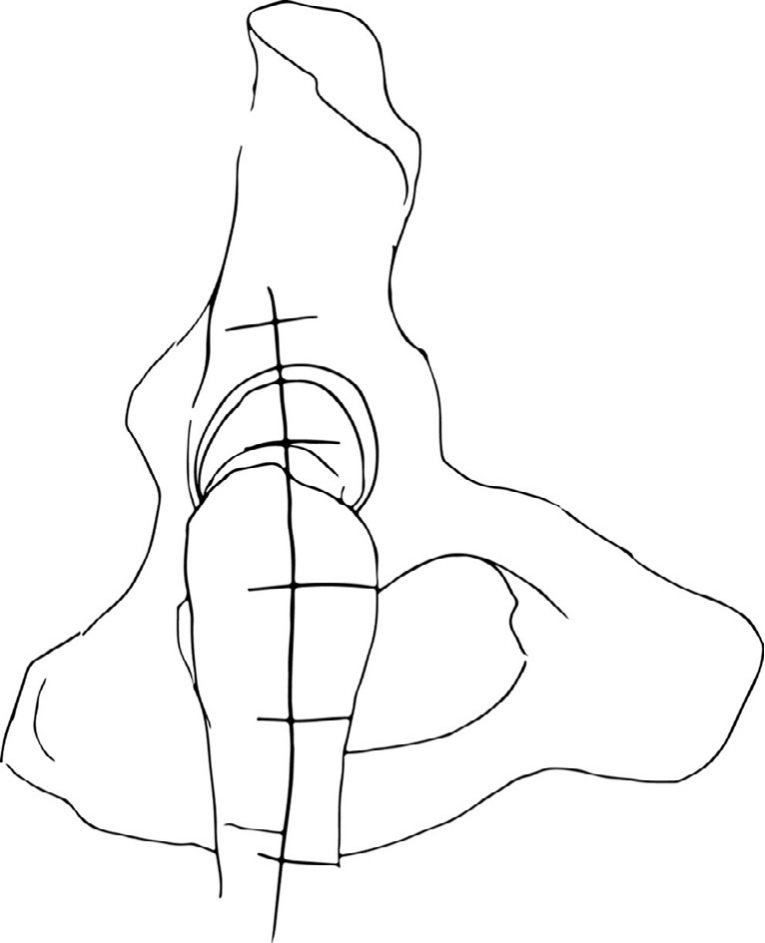
Skin incision drawing.

After skin preparation and draping, a drawing is performed on the skin to determine the accurate positioning incision in the midpart of the GT starting 5 centimeters (cm) proximally to 10 cm distally according to the fracture lines (Figure 2). After the skin incision, *fascia lata* is separated in the same direction between *gluteus medius* and *vastus lateralis*.

**Figure 3.**
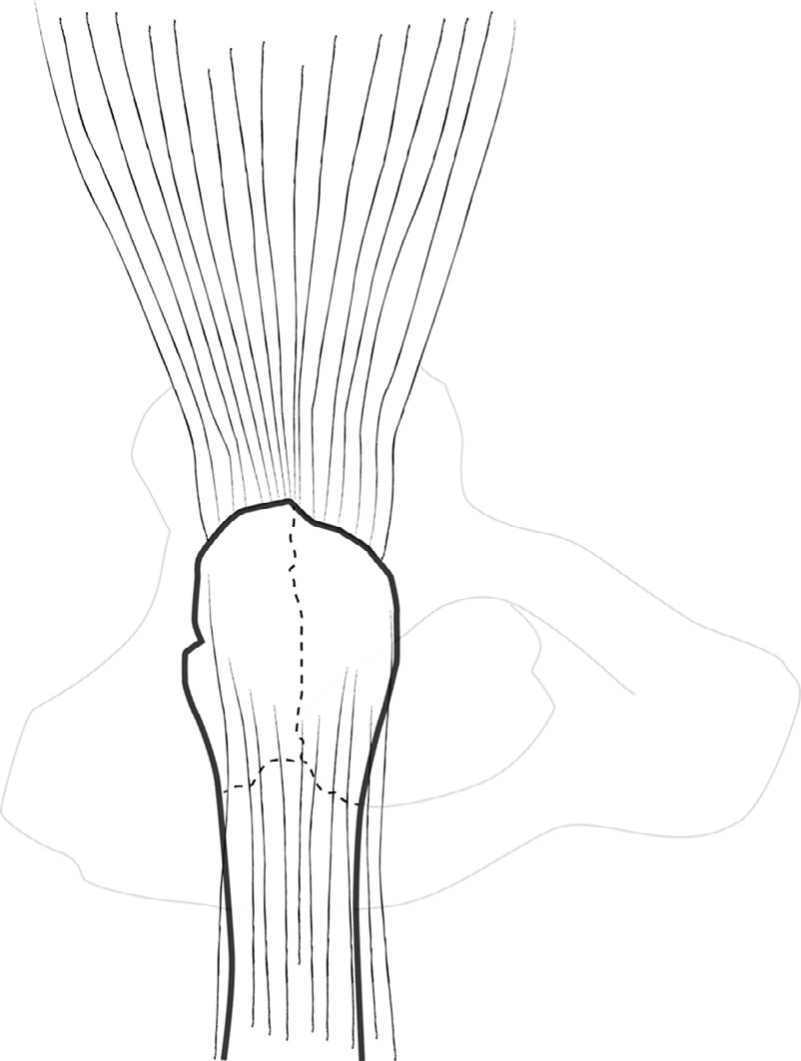
Coronal fractures lines.

**Figure 4.**
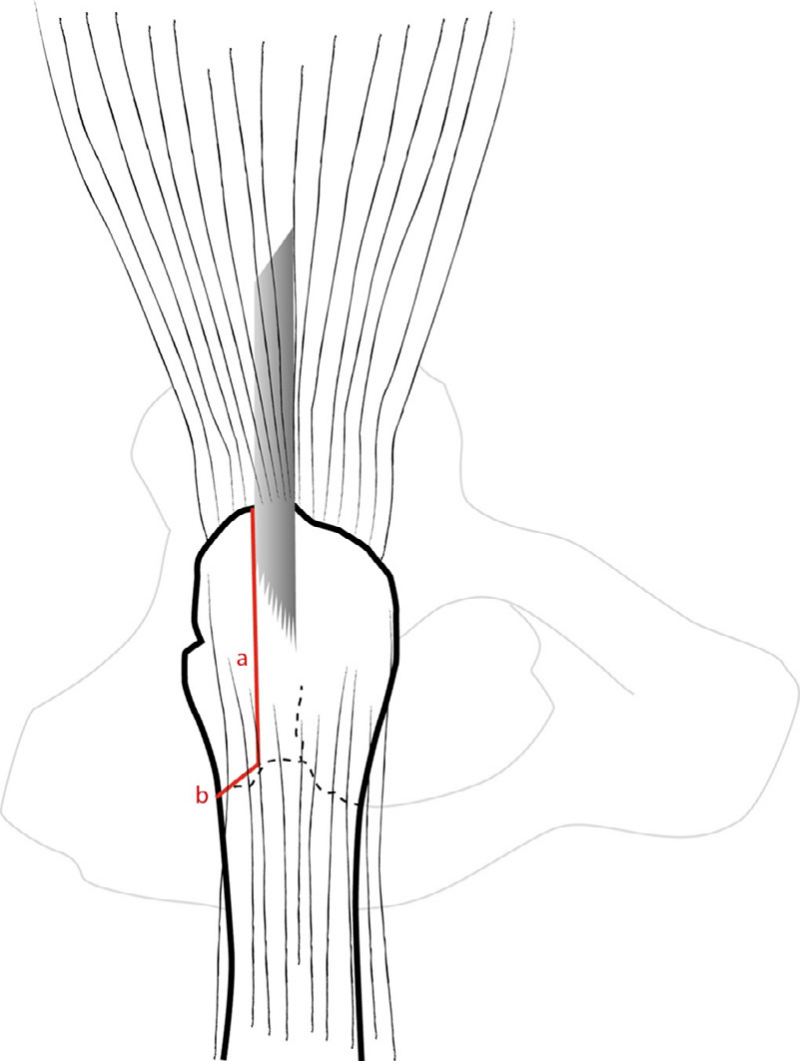
Coronal osteotomy of the great trochanter.

**Figure 5.**
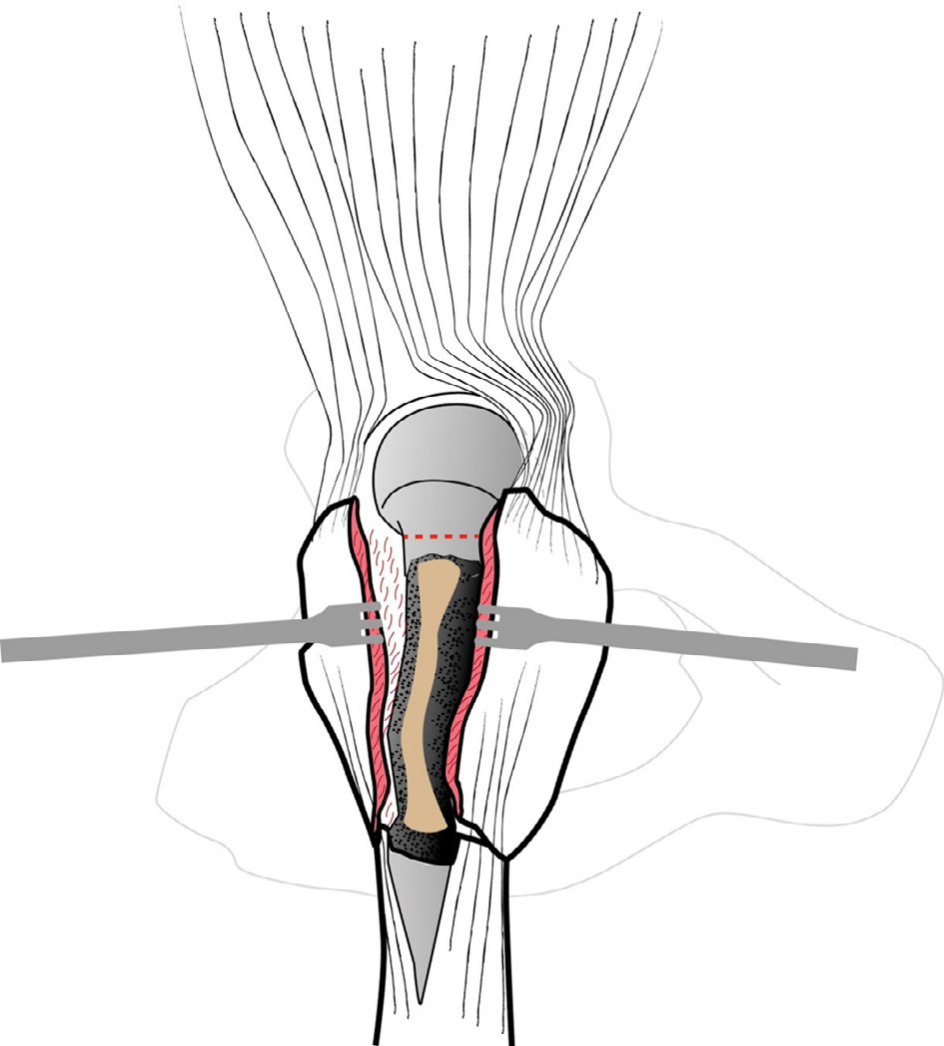
Femoral neck osteotomy (red).

GT and fracture lines are well exposed with retractors. To access and remove the femoral head without internal rotation (and dislocation), separation of proximal fibers of *gluteus medius* tendon is performed over 4–5 cm. If necessary, *vastus lateralis* fibers are separated distally up to the diaphyseal distal fracture line. Unlike Bombaci’s approach (Figure 3) separating each trochanteric piece to access the femoral neck with a small automatic retractor we routinely perform a GT coronal osteotomy (Figure 4) starting at the top of the GT and ending at the trochanteric fossa separating the anterior two thirds from the posterior one third. Two continuous “hemi-digastric” incisions are performed on GT between *gluteus medius*, and *vastus lateralis* to preserve abductor mechanism and allow immediate postoperative full weight bearing. An orthostatic retractor between GT anterior and posterior fragments allows opening the capsule longitudinally. Internal rotation and hip dislocation are not necessary to perform a neck osteotomy with an oscillating saw according to the preoperative planning (Figure 5). Head removal is simple with the help of an extractor corkscrew. Good exposure to perform acetabular preparation is obtained with the help of a McKee retractor.

### Acetabular preparation

**Figure 6.**
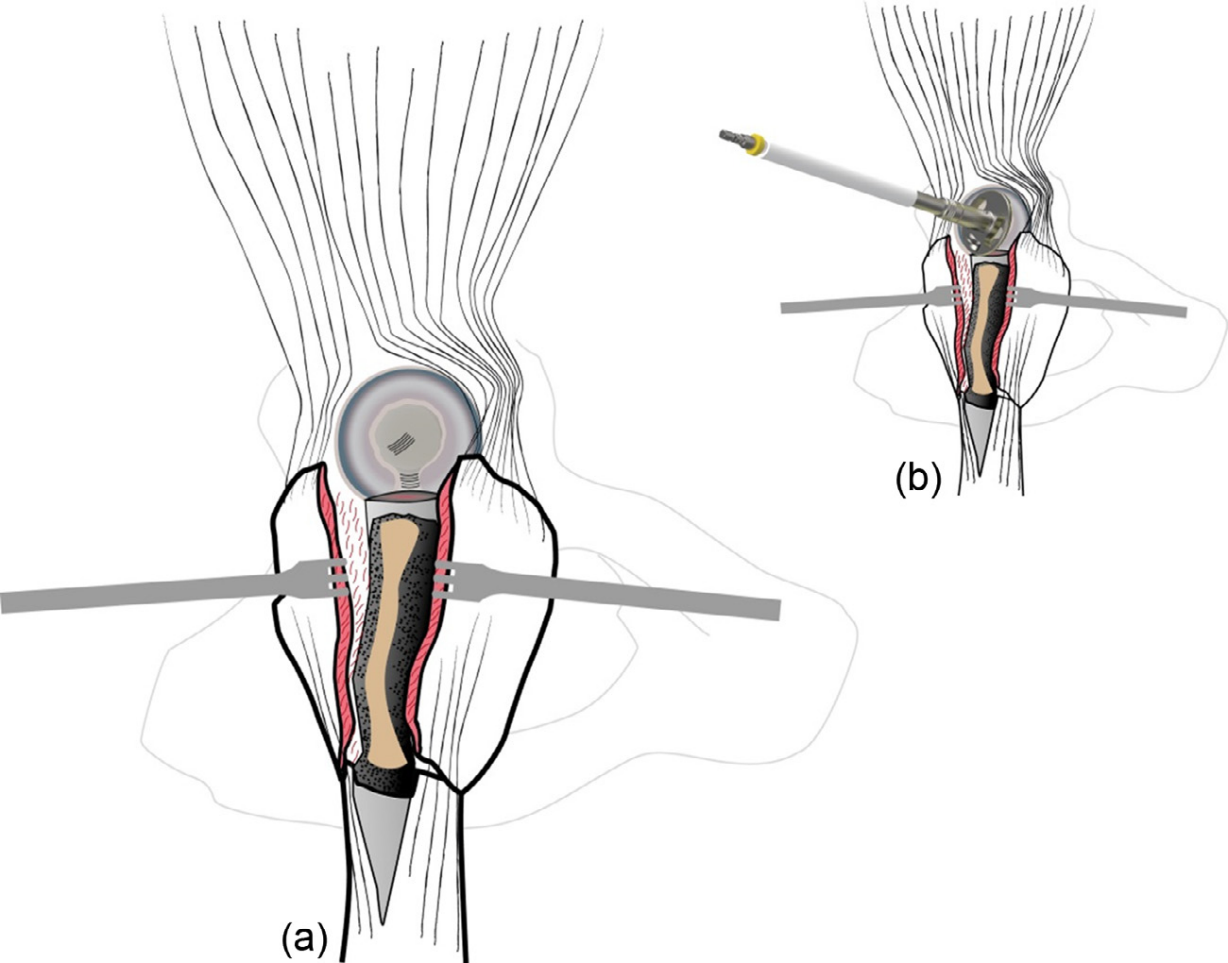
(a) Good exposure of the acetabulum after head removal, (b) reaming.

**Figure 7.**
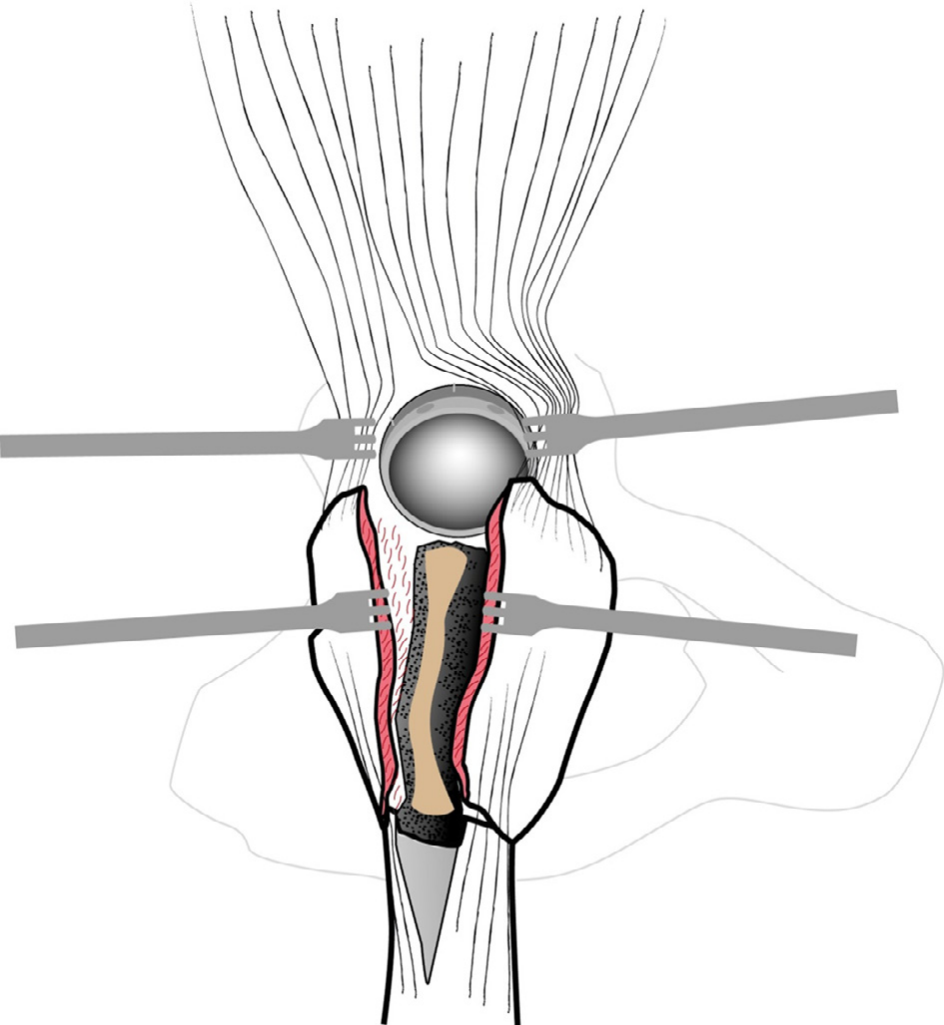
DMC Quattro™ metallic shell.

After resection of the *labrum* and *ligamentum teres*, eventual osteophytes are removed before reaming subchondral bone (Figure 6a). The last reamer size (Figure 6b) is determined by preoperative planning with preservation of the medial wall. After cup trial definitive implant (Figure 7) is inserted. Standard cup as well a dual mobility cup (DMC) could be used; in our experience, we prefer DMC to decrease dislocation risk.

### Femur preparation

Femoral shaft is prepared in the same positional conditions. Proximal canal preparation is first done with crescent size cylindrical reamers in order to avoid any risk of femoral shaft cracks during stem distal cylindrical part impaction. Proximal femur is prepared with rasps according to the preoperative planning. The goal of this last step is to adapt metaphyseal part of the femur to the shape of the implant. Implantation of a trial stem is used in order to:

- check anatomic anteversion,
- adjust leg length according to three parameters:
  - 1 alignment of the head center of trial implant with the top of the GT (like in standard arthroplasty),
  - 2 complete reduction of GT bone fragments around the trial stem,
  - 3 preoperative planning performed on the contralateral hip.

**Figure 8.**
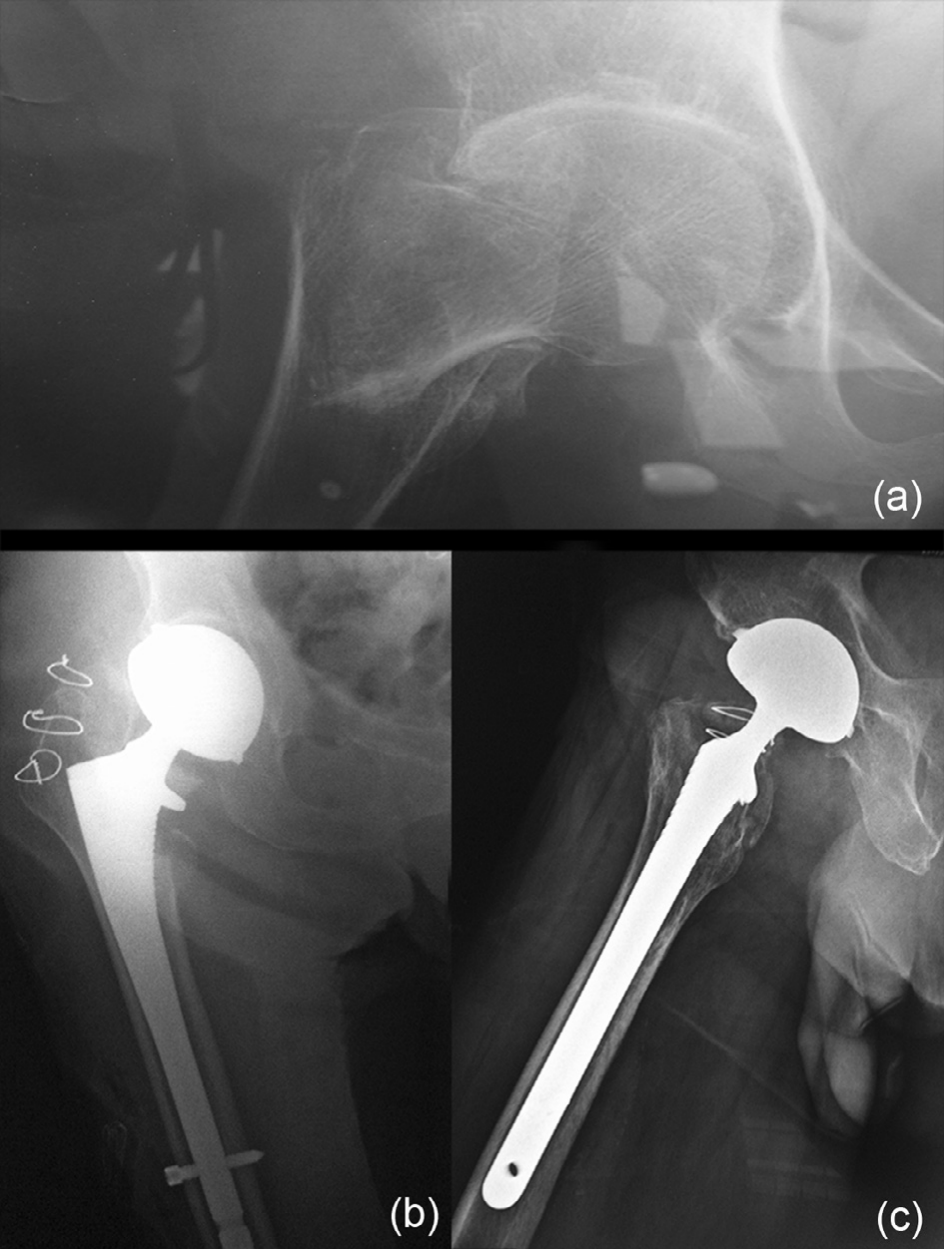
(a) ECTF right hip, (b) cementless stem with distal locking – post-op front view, (c) post-op lateral view.

**Figure 9.**
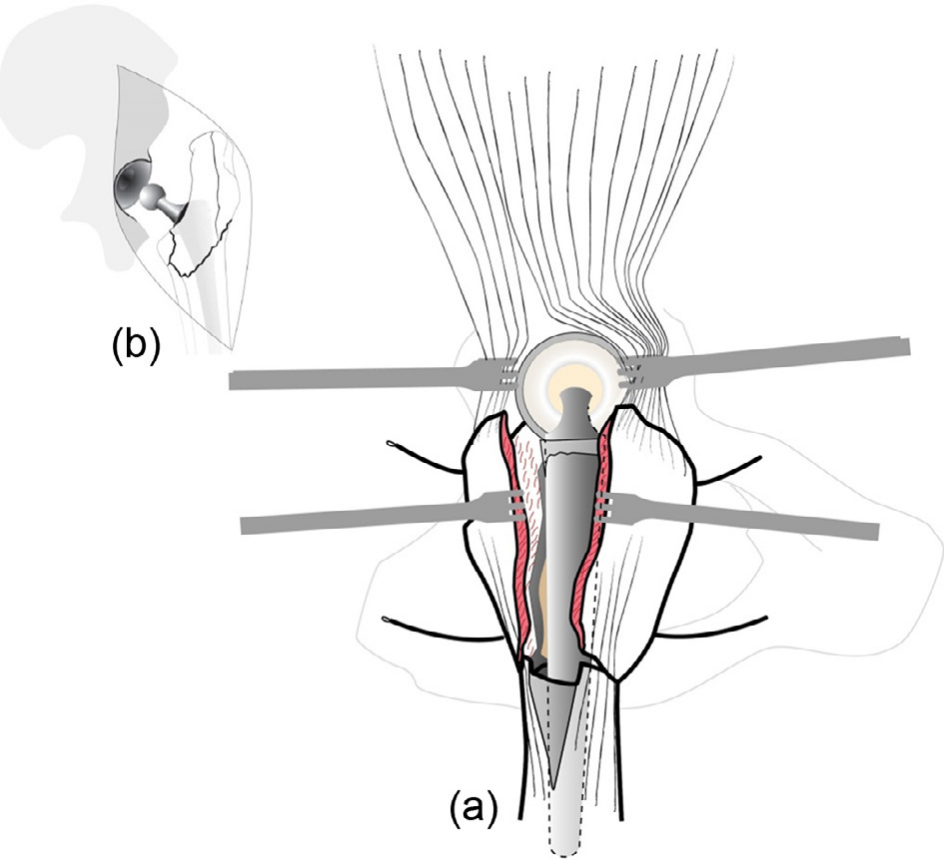
(a) Stem and DMC after reduction, (b) before reduction.

At this stage, to ensure same position of definitive implant, we use a landmark between the top of the extrados and the GT. The cementless stem we routinely use is locked distally to avoid subsidence and/or rotation (Figures 8 and 9).

### Great trochanter and fracture lines fixation

**Figure 10.**
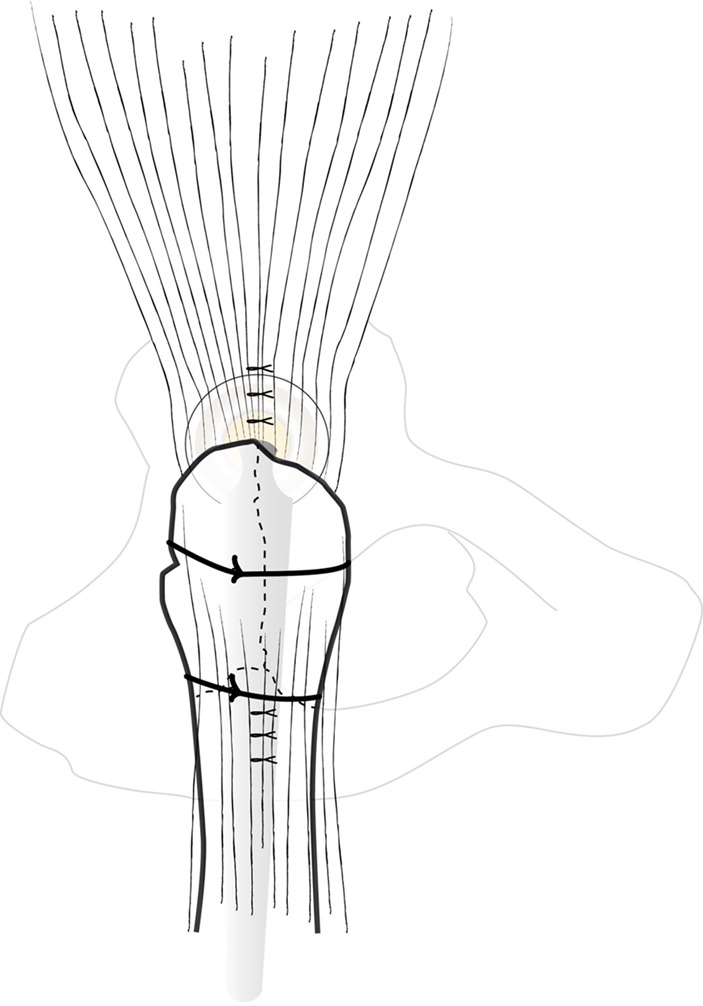
Great trochanter fixation after reattachment with two wires.

THA reduction is performed in the longitudinal axis without rotation of the lower limb. GT is re-attached with one or two crossed standard wires (Figure 10). If fractures lines extend distally, strapping wires around the proximal diaphysis by symmetrical twists complete GT fixation. Hip stability has to be tested.

### Wound closure

The joint is closed by capsular repair if possible; *gluteus medius* and *vastus lateralis* are sutured. *Fascia lata* is repaired and the wound is closed in a regular manner with drainage.

### Postoperative care

Sitting position and full weight bearing are allowed at day one, stair climbing is performed as soon as possible with the help of two crutches.

## Discussion

Unlike the posterior approach, the trans-trochanteric approach (TTA) preserves posterior vascularization (circumflex posterior artery) leading to better bone union conditions. It avoids sciatic nerve injury and preserves external rotator muscles. This procedure could be performed without hip joint dislocation. It allows an anatomical bone repair. The conventional GT osteotomy elevated proximally bone fragments. TTA approach preserves continuity of *gluteus medius*, *vastus lateralis*, and trochanteric area. The lower limb could be maintained in the same position during surgery, helping landmarks and avoiding additional soft tissue lesions. Preservation of abduction mechanism continuity is a guarantee for a quick rehabilitation. Secure fixation of the osteotomy and GT fracture allows better postoperative comfort and quicker rehabilitation.

Compared to Bombaci trans fracture approach the main issue of TTA is adding a coronal osteotomy to a previous complex fracture and could be criticized as an aggravation of initial trauma. Misuse of retractors could also weaken fractured bony pieces. Length adjustment and stem rotation are sometimes difficult to realize due to the lack of precise landmarks. As a last resort numerous wires might be used. On the other hand, exposure is enhanced allowing easier surgery, less surgical time and vascular preservation is essential to ensure GT union.

ECTF are generally treated in France by internal fixation with proximal locking nail or sliding hip screws (90% of ECTF according to French Health Minister data). Operating time is shorter than THA [4] but specific complications are described (nonunion, malunion, length shortening…) and full weight bearing is often hazardous and delayed in elderly patients. The main problem is cutout of the cephalic screw. If osteoarthritis or severe osteoporosis is present, internal fixation could be inappropriate. In case of failure, THA to treat these complications is very difficult and is demanding technique with poor results.

**Figure 11.**
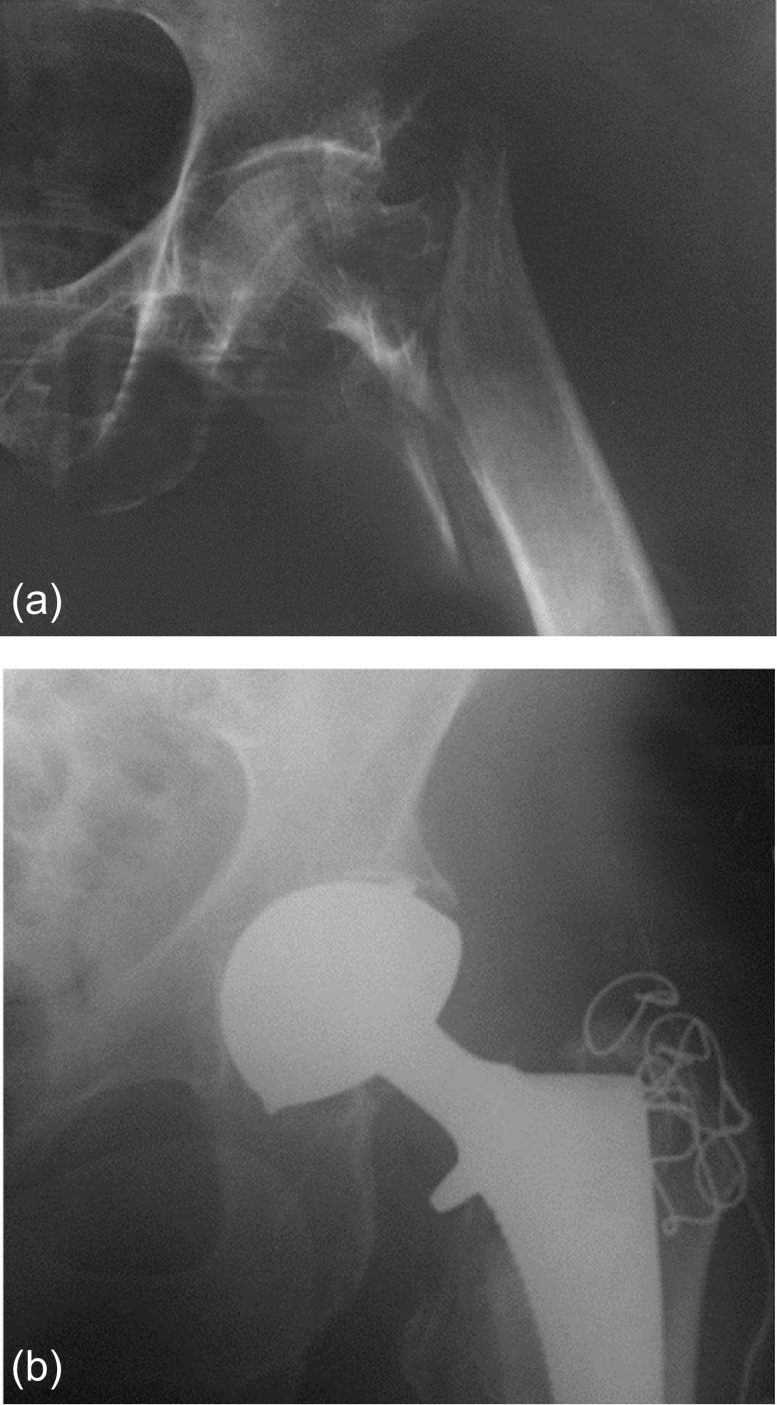
(a) ECTF left hip, (b) treatment by THA with DMC – perfect stability.

Therefore, surgical treatment of ECTF must be a “single shot surgery” including preserving approach, appropriate implant choice, and a stable trochanteric fixation. In our practice, we routinely use a THA with dual-mobility cup to avoid dislocation (Figure 11) that is more frequent with standard ones (5–15%) [5, 6]. We use cementless revision stem with locking device in order to bypass the metaphyseal fracture lines. In case of major osteoporosis and absence of distal fracture lines, cemented stem should be preferred.

This surgical strategy has been applied since 2000 at our trauma center [7]. The rate of readmission is low and consolidation of GT has been always obtained. Patient’s comfort is improved and mortality rate decreased.

## Conclusion

The trans-trochanteric approach may be considered as an improvement on the trans fracture approach initially described by Bombaci [3], enhancing joint exposure, preserving extensor mechanism, and posterior capsule as well as vascularization. It can be used to treat ECTF with joint arthroplasty in the elderly.

## Conflict of interest

François Steffan, Jean-Louis Prudhon, Jean-Marc Puch, André Ferreira, Loys Descamps and Jacques Caton are consultants of groupe Lépine. Régis Verdier is employed by groupe Lépine.

## References

[1] Charnley J (1979) Low friction arthroplasty of the hip. Theory and Practice. Berlin, Heidelberg, New York, Springer.

[2] Caton J, Prudhon JL (2011) Over 25 years survival after Charnley’s total hip arthroplasty. Int Orthop 35, 185–188. 21249358 10.1007/s00264-010-1197-z PMC3032109

[3] Bombaci H (2008) Transtrochanteric approach in intertrochanteric femur fractures. J Trauma 65, 1171–1173. 18753984 10.1097/TA.0b013e318180f58b

[4] Parker MJ, Handoll HHG (2006) Replacement artroplasty versus internal fixation for extracapsular hip fractures in adults. Cochrane Database of Systematic Reviews, Issue 2, CD000086. 16625528 10.1002/14651858.CD000086.pub2 PMC7058115

[5] Caton JH, Prudhon JL, Ferreira A, Aslanian T, Verdier R (2014) A comparative and retrospective study of three hundred and twenty primary Charnley type hip replacements with a minimum follow up of ten years to assess wether a dual mobility cup has a decreased dislocation risk. Int Orthop 38, 1125–1129. 24737147 10.1007/s00264-014-2313-2 PMC4037498

[6] Prudhon JL, Ferreira A, Verdier R (2013) Dual mobility cup: dislocation rate and survivorship at ten years of follow-up. Int Orthop 37, 2345–2350. 24026216 10.1007/s00264-013-2067-2 PMC3843189

[7] Bonnevialle P, Saragaglia D, Ehlinger M, Tonetti J, Maisse N, Adam P, Le Gall C, French H, Knee S, Trauma Surgery A (2011) Trochanteric locking nail versus arthroplasty in unstable intertrochanteric fracture in patients aged over 75 years. Orthop Traumatol Surg Res 97, S95–S100. 21903500 10.1016/j.otsr.2011.06.009

